# Anti-HBV Activities of Three Compounds Extracted and Purified from *Herpetospermum* Seeds

**DOI:** 10.3390/molecules22010014

**Published:** 2016-12-27

**Authors:** Pu-Yang Gong, Zhi-Xiang Yuan, Jian Gu, Rui Tan, Jia-Chuan Li, Yan Ren, Sha Hu

**Affiliations:** 1 College of Pharmacy, Southwest University for Nationalities, Chengdu 610041, Sichuan, China; gongpuyang1990@163.com (P.-Y.G.); ligat@163.com (J.-C.L.); renyan@swun.com (Y.R.); 2 Department of Pharmacy, College of Veterinary Medicine, Sichuan Agricultural University, Chengdu 611130, Sichuan, China; zhixiang-yuan@hotmail.com; 3 College of Medicine, Southwest Jiaotong University, Chengdu 610031, Sichuan, China; tanrui@home.swjtu.edu.cn (R.T.); hs20091688@sina.com (S.H.)

**Keywords:** HepG2.2.15 cells, *Herpetospermum* seeds, hepatitis B virus

## Abstract

The goal of this research was to evaluate the anti-hepatitis B virus (HBV) activities of three compounds extracted and purified from *Herpetospermum* seeds (HS) on HepG2.2.15 cells. Herpetin (HPT), herpetone (HPO), and herpetfluorenone (HPF) were isolated from HS and identified using HR-ESI-MS and NMR. Different concentrations of the drugs were added to the HepG2.2.15 cells. Cell toxicity was observed with an MTT assay, cell culture supernatants were collected, and HBsAg and HBeAg were detected by ELISA. The content of HBV DNA was determined via quantitative polymerase chain reaction (PCR) with fluorescent probes. The 50% toxicity concentration (TC_50_) of HPF was 531.48 μg/mL, suggesting that this species is less toxic than HPT and HPO. HPT and HPF showed more potent antiviral activities than HPO. The 50% inhibition concentration (IC_50_) values of HPF on HBsAg and HBeAg were 176.99 and 134.53 μg/mL, respectively, and the corresponding therapeutic index (TI) values were 2.66 and 3.49, respectively. HPT and HPF were shown to significantly reduce the level of HBV DNA in the HepG2.2.15 culture medium compared to the negative control. This initial investigation of the anti-HBV constituents of HS yielded three compounds that revealed a synergistic effect of multiple components in the ethnopharmacological use of HS.

## 1. Introduction

The hepatitis B virus (HBV) remains a major human pathogen, with more than 240 million individuals experiencing chronic HBV infections [1]. Increasing evidence indicates that persistent HBV infection is associated with end-stage liver diseases, such as cirrhosis and hepatocellular carcinoma (HCC) [2]. The worldwide incidence of HCC ranks fifth out of all malignant tumors, and patients with HCC in China account for more than half of the total cases reported globally [3]. Thus, the prevention and treatment of hepatitis B is very important for reducing the incidence of HCC. Although several antiviral drugs, including interferon-α and nucleoside analogues, have been approved for the treatment of hepatitis B, significant issues remain, including moderate efficacy, dose-dependent side effects, and drug resistance [4]. Therefore, the research and development of new anti-HBV drugs with low toxicity and high performance are imminently needed.

Natural or botanic compounds, unlike nucleoside analogues and interferon-α, have high chemical diversity and biochemical specificity; natural products hold great promise as potentially effective anti-HBV agents. Many phytochemicals, including flavonoids, terpenes, polysaccharides, alkaloids, and lignans, have been isolated and investigated for anti-HBV activities both in vitro and in vivo [5,6]. In China, traditional Chinese medicines (TCMs) containing numerous natural herbs with antiviral and hepatoprotective activities are commonly used as supplementary medicines or as an alternative to interferon-α, with low cost and greater safety [7]. Many anti-HBV candidates with diverse mechanisms and structures have been reported from diverse TCMs, such as astragalosides from *Radix Astragali*, wogonin from *Scutellaria radix*, swerilactones from *Swertia mileensis*, and protocatechuic aldehydes from *Salvia miltiorrhiza* [8,9,10,11]. These promising achievements prompted us to investigate more active compounds from other traditional medicinal herbs to suppress chronic hepatitis B.

*Herpetospermum* seed (HS), a common medication used in Chinese Tibetan medicine, is the dried ripe seed of *Herpetospermum caudigerum* Wall. This species is widely distributed in the southwest region of China, Nepal and the northeast region of India, at an altitude of 2300–3500 m [12]. *H. caudigerum* grows in shrubs and forest margins and along roadsides, and it has a bitter taste. In Tibet, it is a well-known treatment and is used in traditional medicine for the treatment of liver diseases, cholic diseases, and dyspepsia. A preliminary study showed that its chemical constituents mainly include fatty acids, lignans, amino acids and polysaccharides [13]. Extracts of HS have been reported to display a broad spectrum of biological activities, including hepatoprotective, anti-fatigue, anoxia-resistant, and antiviral activities [14,15]. Ganneng Dripping Pill has a significant therapeutic effect in the treatment of chronic hepatitis B, and its main component is lignans from HS [16]. Herpetin (HPT) is a bioactive lignan isolated from HS that reduces the expression and secretion of hepatitis B antigens and HBV DNA. Moreover, HPT can inhibit the proliferation of liver cancer cells in vitro, but the underlying mechanism has yet to be clarified [17]. Furthermore, whether other lignans such as herpetone (HPO) and herpetfluorenone (HPF) from HS have anti-HBV activity remains unknown. 

Therefore, in this study, HPT, HPO, and HPF were isolated and purified from among the lignans of HS, and their in vitro anti-HBV activities were evaluated on the HBV reporter cell line cultured HepG2.2.15.

## 2. Results

### 2.1. Isolation and Identification of HPO, HPT and HPF

**Figure 1:**
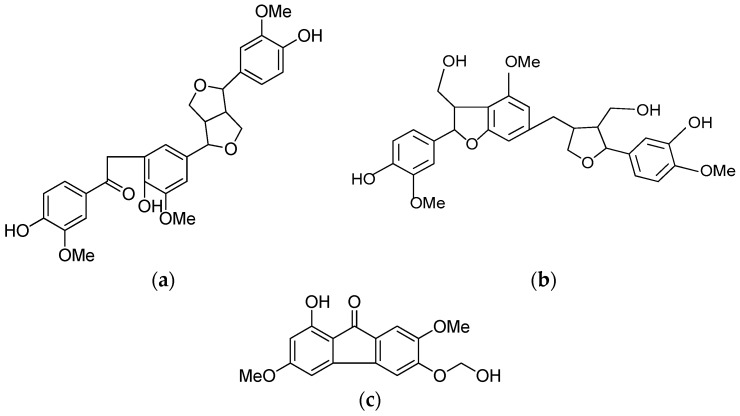
The chemical structures of three lignans isolated from *H.* *caudigerum* seeds (HS). The data obtained here were consistent with previous studies. (**a**) Herpetone (HPO); (**b**) Herpetin (HPT); (**c**) Herpetfluorenone (HPF).

The masses of the petroleum ether, ethyl acetate and *n*-butanol fractions of the ethanol extract of HS were 217, 208 and 107 g, respectively. The ethyl acetate fraction was further purified on a silica gel chromatography column, and HPO was obtained as a pale yellow, oily compound. The structural identification of HPO was as follows: molecular weight: 522 (HR-ESI-MS); molecular formula: C_29_H_30_O_9_. ^1^H-NMR (400 MHz, acetone-*d*_6_) δ: 7.71 (1H, d, *J* = 8.3, 2.0 Hz, H-6), 7.65 (1H, d, *J* = 1.6 Hz, H-2′), 6.98 (1H, d, *J* = 1.7 Hz, H-2′′), 6.89 (1H, d, *J* = 8.2 Hz, H-5), 6.89 (1H, d, *J* = 1.8 Hz, H-6′), 6.83 (1H, dd, *J* = 8.1, 1.7 Hz, H-6′′), 6.81 (1H, d, *J* = 2.1 Hz, H-2), 6.78 (1H, d, *J* = 8.1 Hz, H-5′′), 4.65 (2H, d, *J* = 4.6 Hz, H-7′, 7′′), 4.22 (2H, s, H-8), 4.15~4.23 (2H, m, H-8′, 8′′), 3.88 (3H, s, -OCH3), 3.84 (3H, s, -OCH3), 3.83 (3H, s, -OCH3), 3.79 (1H, m, H-8′), 3.77 (1H, m, H-8′′). The structure of HPO was confirmed and is shown in Figure 1. The results are consistent with those of a previous study by [13].

To efficiently isolate the target compound, silica gel column chromatography using a methanol-chloroform gradient system was applied to separate the ethyl acetate fraction into 18 subfractions. According to the pre-experimental results, the 10th subfraction was further isolated to yield 1.19 g of HPT powder as a white amorphous solid. The structural identification of HPT was achieved using HR-ESI-MS and ^1^H-NMR, as follows: HR-ESI-MS *m*/*z*: 561.21601 [M + Na]^+^, molecular formula: C_30_H_34_O_9_. ^1^H-NMR (400 MHz, acetone-*d*_6_) δ: 7.63 (1H, s, 4-OH), 7.46 (1H, s, 3′′-OH), 7.04 (1H, d, *J* = 1.8 Hz, H-2), 6.94 (1H, d, *J* = 1.5 Hz, H-2′′), 6.89 (1H, dd, *J* = 8.2, 1.8 Hz, H-6), 6.80 (1H, d, *J* = 8.1 Hz, H-5), 6.76~6.78 (3H, m, H-6′, 5′′, 6′′), 6.74 (1H, br s, H-2′), 5.52 (1H, d, *J* = 6.7 Hz, H-7), 4.78 (1H, d, *J* = 6.2 Hz, H-7′′), 4.12 (1H, m, H-9′), 3.97 (1H, dd, *J* = 8.6, 6.2 Hz, H-9′), 3.85 (2H, m, H-9′′), 3.83 (3H, s, 3-OCH3), 3.81 (6H, s, 3′, 4′′-OCH3), 3.68 (1H, dd, *J* = 8.0, 6.8 Hz, H-9), 3.52 (1H, dd, *J* = 12.6, 6.5 Hz, H-8′′), 2.97 (1H, m, H-7′), 2.71 (1H, m, H-8), 2.55 (1H, m, H-7′), 2.31 (1H, m, H-8′). The data presented above agree with previously published values [17] obtained from a study in which a white amorphous powder was identified as HPT. To obtain purified HPF, the sixth subfraction was further isolated to yield HPF powder as a white amorphous solid using Sephadex LH-20 gel column chromatography. The structural identification of HPF obtained by HR-ESI-MS and 1H-NMR spectra was as follows: molecular weight: 302; molecular formula: C_16_H_14_O_6_. ^1^H-NMR (acetone-*d*_6_, 400 MHz) δ: 8.19 (1H, s, H-8), 7.70 (1H, s, H-5), 7.07 (1H, d, *J* = 2.8 Hz, H-4), 6.85 (1H, d, *J* = 2.8 Hz, H-2), 5.02 (2H, s, -OCH2-), 4.05 (3H, s, 7-OCH3), 3.90 (3H, s, 3-OCH3). These data were consistent with previous studies.

### 2.2. Cytotoxic Effects of Drugs on HepG2.2.15 Cells

**Figure 2:**
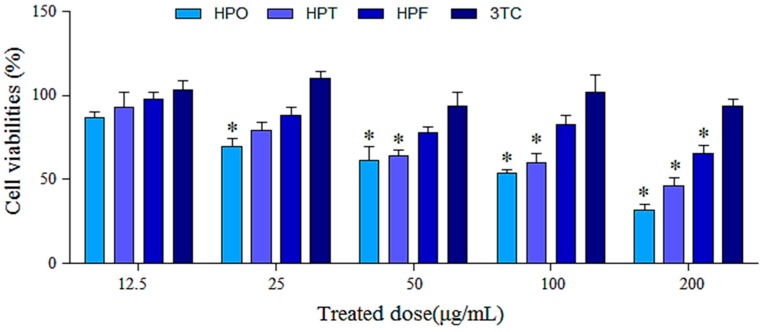
Inhibitory effects of HPO, HPT, HPF and 3TC (12.5, 25, 50, 100, and 200 μg/mL) on HepG2.2.15 cells. The proliferation of HepG2.2.15 cells was measured via MTT assay. Values are expressed as the means ± SD (*n* = 4). * *p* < 0.01 vs. (3TC) control.

The viability of HepG2.2.15 cells in the presence of different concentrations (12.5, 25, 50, 100, and 200 μg/mL) of HPO, HPT, HPF and 3TC was determined using an MTT assay. After six days of treatment with the different compounds, we observed that HPO and HPT inhibited the proliferation of HepG2.2.15 cells in a concentration-dependent manner. As shown in Figure 2, HPF showed lower toxicity in HepG2.2.15 cells at various concentrations than HPO and HPT did. When the concentration of HPO was 200 μg/mL, the highest suppression ratio was 68.29% in these cells. No significant cytotoxicity was exhibited by 3TC compared to the negative control at concentrations of 12.5–200 μg/mL. As shown in Table 1, the 50% toxicity concentration (TC_50_) values of the drugs were detected to determine the concentrations to use in subsequent experiments.

### 2.3. Inhibitory Effects of Three Compounds and 3TC on HBV Antigens

HBsAg and HBeAg are important antigens in the infection and replication of HBV. Higher antigen titers reflect more serious infections. Thus, we investigated the inhibitory effects of these drugs on antigens used as standards for detecting compounds’ anti-HBV activities. The supernatant of the MTT assay was collected to detect the effects of compounds on HBV antigen secretion in HepG2.2.15 cells. 

**Figure 3:**
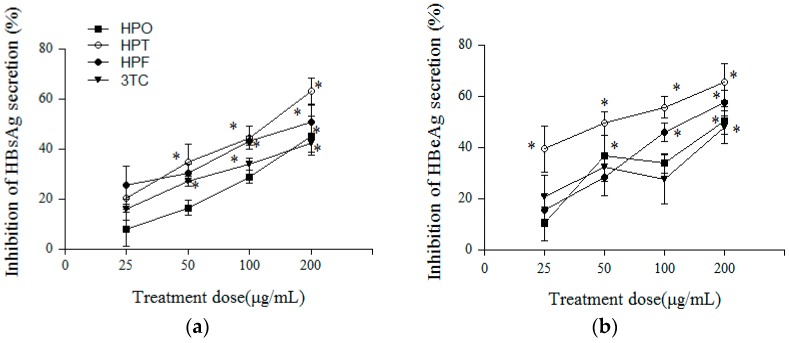
Inhibitory effects of HPO, HPT, HPF and 3TC on HBsAg and HBeAg secretion from HepG2.2.15 cells. HepG2.2.15 cells were treated with HPO, HPT, HPF and 3TC (25, 50, 100, and 200 μg/mL) in 96-well culture plates at 37 °C in 5% CO_2_. After six days, the levels of (**a**) HBsAg and (**b**) HBeAg in the culture supernatants were detected with commercial ELISA kits. * *p* < 0.01 vs. (3TC) control.

The inhibitory effects of HPO, HPT, HPF, and 3TC on HBV antigens are summarized in Figure 3. HPT was the most potent inhibitor of HBsAg and HBeAg secretion, followed by HPF. When the concentration of HPT was 200 μg/mL, the highest suppression ratio (65.5%) for HBeAg in the supernatant was obtained (Figure 3). To clarify whether the antigen-inhibitory effect of a drug depends on high toxicity, the therapeutic index (TI) is calculated. A TI above 2 indicates that a drug is significantly effective and has low toxicity (Table 1). HPF more efficiently inhibited HBeAg secretion by HepG2.2.15 cells at the six-day time point than the other compounds, and its TI was 3.49. Additionally, we observed that HPT and HPF inhibited HBsAg and HBeAg secretion by HepG2.2.15 cells in a concentration-dependent manner and exhibited TI values above 2. In contrast, after six days of treatment, HPO had no significant inhibitory effects on the secretion of HBsAg and HBeAg in the culture supernatant, and its TI was below 1 because of its high cytotoxicity.

### 2.4. Effects of Three Compounds and 3TC on the Secretion of HBV DNA

**Figure 4:**
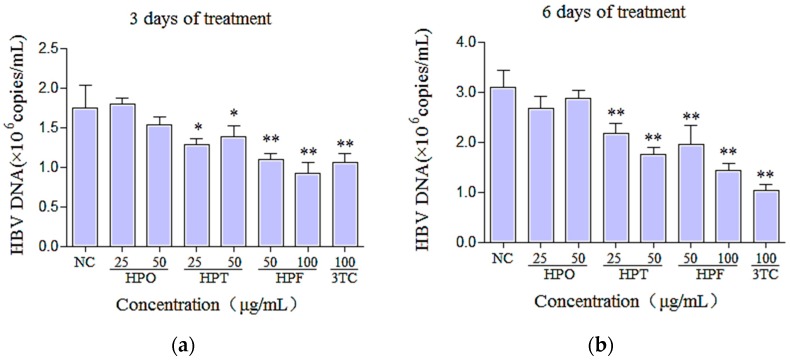
Inhibitory effects of HPO, HPT, HPF and 3TC on the expression of HBV DNA in the supernatant. HepG2.2.15 cells were treated in the presence of different concentrations of HPO, HPT, or HPF or 100 μg/mL 3TC for (**a**) three days and (**b**) six days. The levels of HBV DNA in the supernatant were quantified using reverse transcriptase (RT)-PCR. A negative control (with no drug treatment) was treated with vehicle. The results are presented as the means ± SD. ** *p* < 0.01, * *p* < 0.05 vs. negative control.

HBV is a DNA virus, and the content of HBV DNA in the supernatant reflects the reproductive ability of HBV. To further test the potential anti-HBV activities of the three new compounds in HepG2.2.15 cells, the effect of the drugs on the HBV DNA content in the supernatant was evaluated with non-cytotoxic concentrations of the drugs; the results are shown in Figure 4. After treatment for three days, the HBV DNA content in the supernatants of the HPT- and HPF-treated groups declined. The HPO-treated group did not differ significantly from the negative control (*p* > 0.05). After the cells were treated for six days, HPT and HPF significantly reduced the replication of HBV DNA in the medium compared to the negative control group (*p* < 0.01). At the same time point, 100 μg/mL HPF inhibited HBV DNA replication by 53.87%. HPF appeared to be the most effective, whereas HPO was not found to exert a significant inhibitory effect on HBV DNA at concentrations of 25 and 50 μg/mL. The positive control 3TC (100 μg/mL) reduced the content of HBV DNA secreted by HepG2.2.15 cells into the medium after three and six days.

## 3. Discussion

Substantial effort has been exerted to find antiviral agents in natural sources. Many plants and herb species have been reported to have potential antiviral activities, and a wide variety of active compounds have been identified from plants. In China, many bioactive compounds from traditional herbs or their derivatives have been widely used in the treatment of viral hepatitis and associated complications, such as liver cirrhosis and liver failure [18,19]. In this study, three lignan compounds (HPO, HPT, and HPF) were extracted and purified from HS according to previously reported methods. HPT and HPF were isolated from HS using Sephadex column chromatography with some improvements. HR-ESI-MS and NMR spectra were used to identify the structures of the obtained compounds, which were consistent with published reports.

Lignans from Tibetan medicinal HS were previously reported to exert a certain hepatoprotective effect on ConA-induced immunological liver injury in mice, which may be related to their antioxidant and anti-inflammatory activities [20]. Lignans extracted from Tibetan medicinal HS also possess some protective effects against carbon tetrachloride–induced acute liver injury in mice, and the protective mechanism also included in inhibiting the expression of inflammatory factors [21]. To some extent, the pathogeneses of immunological liver injury and hepatitis are similar. Therefore, numerous experiments were conducted to determine whether HS exerts a protective anti-HBV effect. HPT extracted from HS was previously reported to show anti-HBV properties in vitro, and a clinical trial on the anti-HBV effects of an HS extract in human subjects is currently ongoing [16]. However, whether the other lignan compounds in HS possess similar properties has not been determined. Furthermore, little attention has been paid to anti-tumor bioactivities. In this study, the HBV-positive HepG2.2.15 cell line, which secretes HBsAg, HBeAg and HBV DNA into the medium, was used to investigate the anti-HBV effects mediated by three compounds derived from HS. 

As indicated by the MTT results, the TC_50_ of HPF was 469.92 μg/mL, which suggested that HPF is less toxic than HPO and HPT. The MTT assay is typically used for screening anti-tumor drugs, and the results of this study demonstrated that HPO and HPT had potential anti–liver cancer effects. The same concentrations used for the HBsAg and HBeAg experiments were used to calculate the TI. HPF significantly inhibited the secretion of HBsAg and HBeAg, and as the concentration of HPF increased, a dose-dependent response was observed. The IC_50_ values of HPF in HepG2.2.15 cells based on HBsAg and HBeAg were 176.99 and 134.53 μg/mL, respectively, and the TI values were 2.66 and 3.49. HPF also exerted a significant inhibitory effect on HBV DNA. HPF was shown to be more potent and effective than the other two compounds. Additionally, the TI values of HPT based on HBsAg and HBeAg were 1.28 and 2.54, respectively, suggesting that HPT showed a significant inhibitory effect against HBeAg and had low toxicity. The data from these experiments showed that not only did single components extracted from HS possess antiviral effects, but multiple components also exerted a synergistic effect. The inhibitory effects of HPO could be attributed to the reduction in cell proliferation resulting from this compound’s high toxicity in HepG2.2.15 cells. Thus, we plan to study the anti-tumor effects of these compounds from HS in the next stage of our research.

Chronic HBV infection, which is a major risk factor for HCC, was reported to be associated with more than half of HCC cases worldwide [22,23]. The HBV X protein (HBx) has been reported to play an important role in the development of HCC by influencing the cell cycle, proliferation, and apoptosis at the level of cell signaling and transcription [24]. HBV also plays a direct role in liver transformation by triggering both common and etiology-specific oncogenic pathways, stimulating the host immune response, and driving chronic liver necroinflammation [25]. Recently, Liu and colleagues found that HBeAg and its precursors promoted HDM2-mediated degradation and impaired the transcriptional activity of p53 by interacting with NUMB, which contributed to HCC development [26]. In summary, the inhibition of HBV is essential to prevent the development of liver cancer. Additionally, searching for anti-HBV and anti-tumor drugs from natural plant sources is one research direction that can be pursued. The results of this study demonstrated that HPT and HPF significantly suppressed HBeAg secretion into the medium. HPT and HPF may be able to prevent the occurrence of liver cancer to a certain extent. We also observed the effects of the three compounds on tumor resistance through an MTT assay. We speculate that these drugs could affect the viability of liver cancer cells. This study showed that the three compounds from HS have certain anti-HBV effects and could contribute to the prevention of liver cancer.

However, we could not identify whether other lignan compounds also possess anti-HBV and anti-tumor activities because we only evaluated the activities of three of these compounds. More lignan compounds need to be isolated and purified from this herb to screen their biological activity. Based on the efficacy of TCMs used in multi-component and multi-target preparations, the anti-HBV activities of combinations of compounds should also be evaluated. In addition, determining the precise mechanisms underlying the anti-HBV effect of the HS extract requires additional investigation. For example, the effects of HPT and HPF on the activities of HBV promoters that act as molecular switches and determine gene activity could be elucidated.

This study clearly demonstrated that compounds isolated and purified from the lignan fraction of HS exhibited dose-dependent antiviral activities in HepG2.2.15 cells (HPF showed activity inhibiting HBsAg secretion; HPT and HPF exhibited activity inhibiting HBeAg secretion; and HPF showed significant activity inhibiting HBV DNA replication). Thus, these results will provide valuable support for the use of constituents extracted from HS as novel anti-HBV agents.

## 4. Materials and Methods

### 4.1. Materials

Silica gel and Sephadex LH-20 were purchased from Sigma-Aldrich Company Ltd. (Gillingham, UK). Dimethyl sulfoxide (DMSO), 3-(4,5-dimethylthiazol-2-yl)-2,5-diphenyltetrazolium bromide (MTT), and G418 were purchased from Sigma (St. Louis, MO, USA). The enzyme-linked immunosorbent assay (ELISA) kit for HBsAg and HBeAg was obtained from the Beijing Wantai Biological Pharmacy Enterprise Co., Ltd. (Beijing, China). The HBV DNA extraction and amplification fluorescence assay kit was purchased from the Sansure Biotechnology Company (Changsha, China). RPMI 1640 medium, trypsin, ethylenediaminetetraacetic acid (EDTA), and l-glutamine were obtained from Gibco-BRL (Carlsbad, CA, USA). The compound 3TC was purchased from GlaxoSmithKline Pharmaceuticals Ltd. (Suzhou, China) and was freshly prepared before each use. The chemical reagents used for extraction and purification were of analytical grade and were purchased from Nanjing Chemical Reagent Co., Ltd. (Nanjing, China).

### 4.2. Plant Material

HS were purchased from the Hehuachi medicinal material market (Chengdu, China) in June 2015 and were authenticated by Prof. Liang-Ke Song at the College of Medicine, Southwest Jiaotong University. A voucher specimen (no. 2015100911) was stored at the herbarium of the College of Pharmacy, Southwest University for Nationalities. 

### 4.3. Isolation and Identification of Compounds

HPO, HPT and HPF were obtained according to previously published methods with some improvements [12,13]. Briefly, 7.0 kg of dried and powdered HS was extracted twice with 6 L of 80% ethanol solution for 2 h, and then the ethanol was evaporated. The extract was partitioned into petroleum ether, ethyl acetate and *n*-butanol parts. Then, chloroform:methanol (99:1) was used to elute the ethyl acetate fraction via silica gel column chromatography, and the resulting fraction was purified with petroleum ether:acetone (1:1) to obtain HPO. The ethyl acetate fraction was concentrated and separated into 18 fractions (Fr. 1–Fr. 18) via silica gel column chromatography using a methanol-chloroform gradient system. Then, Fr. 10 was further isolated to obtain crude HPT through silica gel column chromatography eluted with a petroleum:chloroform:acetone (1:1:1) system. The crude HPT was finally purified with a Sephadex LH-20 column with methanol to yield pure HPT. Fr. 6 was further isolated to obtain crude HPF through silica gel column chromatography eluted with a petroleum:chloroform:acetone (1:1:1) system. Then, the resulting product was merged and separated into 12 fractions (Fr. 6.1–Fr. 6.12). Fr. 6.6 was finally purified with a Sephadex LH-20 column and methanol to yield pure HPF. Then, NMR (Bruker Avance III 600 MHz spectrometer) and MS (Bruker Amazon SL, Bremen, Germany) were used for the identification of HPO, HPT, and HPF.

### 4.4. Cell Culture and Drug Treatment

HepG2.2.15 cells from a human HepG2 cell line transfected with the HBV genome were obtained from the State Key Laboratory of Virology at the School of Medicine of Wuhan University and were maintained in RPMI-1640 supplemented with 10% (*v*/*v*) foetal calf serum, 100 U/mL penicillin/streptomycin and 200 μg/mL G418 at 37 °C in a thermostatic and sealed incubator with 5% CO_2_. HPT, HPO and HPF were each dissolved in DMSO and stored at −20 °C until needed. The concentration of DMSO in the culture was lower than 2.5 μL/mL, at which the growth of cells was not affected [27]. To determine the activities of HPT, HPO and HPF, well-grown HepG2.2.15 cells were digested with 0.25% trypsin and seeded in 96-well or 24-well flat-bottom cell culture plates, and the control and treatment groups for different drug treatments were separated.

### 4.5. MTT Assay

HepG2.2.15 cells were seeded in a 96-well plate at a density of 2 × 10^4^ cells/well with 200 μL of RPMI-1640. After incubation for 24 h, the culture medium was replaced with 200 μL of HPT, HPF, or HPO (12.5, 25, 50, 100, and 200 μg/mL) or 3TC (12.5, 25, 50, 100, and 200 μg/mL) in RPMI-1640 medium. Then, the plates were returned to the incubator for six days, and the medium (containing drug) was changed every three days. After six days, the supernatant was saved for HBsAg and HBeAg assays, and 10 μL of MTT (5 mg/mL) was added to each cell well and incubated for 4 h at 37 °C. After the MTT was aspirated, 150 μL of DMSO was added to each well, followed by shaking for 10 min. The optical density (OD) at 570 nm was measured using a DG3022-A automatic microplate reader (Rayto, Shenzhen, China). The cell growth ability was expressed as a percentage of the control.

### 4.6. Determination of HBeAg and HBsAg

After the cells were treated for six days, the levels of HBeAg and HBsAg were measured in the culture medium using an ELISA kit according to the manufacturer’s instructions.

### 4.7. Determination of HBV DNA in the Supernatant

HepG2.2.15 cells (3 × 10^5^ per well) were seeded in a 24-well plate and treated with HPO or HPT (25 and 50 μg/mL), HPF (50 and 100 μg/mL) or 3TC (positive control; 100 μg/mL) for six days. The HBV DNA in the culture supernatants was measured with an HBV DNA polymerase chain reaction (PCR)-fluorescence quantitation kit as follows: HBV DNA was extracted according to the manufacturer’s instructions. The forward primer was 5′-TTCCTCTKCATCCTGCTGC-3′, the reverse primer was 5′-ACAAACGGGCAACATACCTTG-3′, and a TaqMan probe FAM-TATGCCTCATCTTCTTRTTGGTT was used. Samples were amplified and detected with a Bio-Rad CFX96 Real-Time PCR Detection system (Hercules, CA, USA). The cycling program was as follows: 50 °C for 2 min; 94 °C for 5 min; 45 cycles at 94 °C for 15 s and 57 °C for 30 s; and 25 °C for 10 s.

### 4.8. Statistical Analysis

The data from the experiments in this study are expressed as the means ± standard deviation (SD) and were analyzed with the SPSS 15.0 statistical package (SPSS Inc., Chicago, IL, USA). Multiple comparisons were performed with an analysis of variance (ANOVA). A value of *p* < 0.01 was considered statistically significant.

## Figures and Tables

**Table 1:** TI values of three lignans and 3TC for HBsAg and HBeAg secretion from HepG2.2.15 cells.

Compound	TC_50_ (μg/mL)	HBsAg		HBeAg	
		IC_50_ (μg/mL)	TI	IC_50_ (μg/mL)	TI
HPT	146.74	114.48	1.28	57.75	2.54
HPO	91.15	240.31	0.38	185.64	0.49
HPF	469.92	176.99	2.66	134.53	3.49
3TC	-	305.38	-	312.00	-

Hepatitis B (HBsAg) surface antigen; hepatitis B (HBeAg) antigen; Herpetin (HPT); Herpetone (HPO); Herpetfluorenone (HPF); Lamivudine (3TC); 50% toxicity concentration (TC_50_); 50% inhibition concentration (IC_50_); therapeutic index (TI).
